# Fostering collaboration: a controlled pilot study of interprofessional education for medical and pharmacy students

**DOI:** 10.1186/s12909-025-07424-z

**Published:** 2025-06-03

**Authors:** Maike Petersen, Sabine Gehrke-Beck, Nicole Zimmermann, Amrei-Pauline Konrad, Ronja Behrend, Harm Peters, Martin Schulz, Charlotte Kloft, Wolfram J. Herrmann

**Affiliations:** 1 Institute of General Practice and Family Medicine, Charité– Universitätsmedizin Berlin, Charitéplatz 1, 10117 Berlin, Germany; 2 Institute of Pharmacy, Freie Universität Berlin, Kelchstr. 31, 12169 Berlin, Germany; 3 Dean’s Office for Student Affairs, Charité– Universitätsmedizin Berlin, Charitéplatz 1, 10117 Berlin, Germany; 4 Dieter Scheffner Center for Medical Education and Educational Research, Dean’s Office for Student Affairs, Charité– Universitätsmedizin Berlin, Charitéplatz 1, 10117 Berlin, Germany; 5 Department of Medicine, ABDA– Federal Union of German Associations of Pharmacists, Heidestraße 7, 10557 Berlin, Germany

**Keywords:** Interprofessional education, Interprofessional collaboration, Medical students, Pharmacy students

## Abstract

**Background:**

An increasingly ageing population and the resulting multimorbidity is a growing challenge for healthcare systems. A solution to tackle this problem is interprofessional collaboration. Interprofessional education, as an early meeting point, could foster a future successful interprofessional collaboration. So, the aim of our pilot study was to assess whether the “interprofessional collaboration between medical and pharmacy students to improve medication safety in polypharmacy” (PILLE) project changed the perception of medical and pharmacy students towards interprofessional collaboration.

**Methods:**

PILLE consisted of three parts: (1) A mandatory 90-minutes interprofessional seminar; (2) A voluntary 120-minutes practical training; (3) A voluntary half-day interprofessional tandem work shadowing at a General Practitioner’s (GP) office. We used a controlled study design. Medical and pharmacy students from the intervention group attended the seminar (1). Medical students of the control group attended an interactive 90-minutes monoprofessional seminar only. Working together on cases was the similarity of both seminars. The students from the intervention group could attend (2) and (3). The outcome of interest was the change in students’ perception towards interprofessional collaboration after participating in PILLE, using pre- and post-questionnaires with the validated German version of Student Perceptions of Physician-Pharmacist Interprofessional Clinical Education (SPICE-2D) instrument. Descriptive and statistical evaluation was conducted. To assess if there is a significant pre-post-difference in the SPICE-2D overall score within the individual groups, paired t-tests were executed. To investigate if there is a significant change in SPICE-2D overall score difference between the medical students control and intervention group, an unpaired t-test was performed.

**Results:**

In total, 436 students participated: 96 medical students and 116 pharmacy students in the intervention group, and 224 medical students in the control group. The participating students from both professions already had a pronounced positive perception at baseline. All pre-post-mean-differences within the individual groups and between the medical students control and intervention group were not statistically significant.

**Conclusion:**

Participating medical and pharmacy students already had a positive perception at baseline. A one-time and short-term mandatory seminar of 90-minutes seems not to be sufficient to measure a change in perception. Future interprofessional education projects should focus on frequent meeting points between students.

## Background

An increasingly ageing population increases the demand for healthcare [1]. Multimorbidity, for example, is a growing challenge for healthcare systems [2] and in the management of multimorbidity multiple healthcare professionals are involved. In Germany, 42% of patients older than 65 years take five or more chronic medications, stated as polypharmacy [3]. The World Health Organization described in their Framework for Action on Interprofessional Education and Collaborative Practice that “collaborative practice strengthens health systems and improves health outcomes” [4]^(p7)^ In the report, interprofessional collaboration is defined as “collaborative practice happens when multiple health workers from different professional backgrounds work together with patients, families, carers and communities to deliver the highest quality of care” and is seen as one strategy to reduce the healthcare professional shortages around the world. [4]^(p7)^ An example that demonstrates interprofessional collaboration improves patient outcomes is the interprofessional medication management programme ARMIN. Patients had a 16% lower relative risk in all-cause mortality after receiving care from teams of community pharmacists (CP) and general practitioners (GP) [5]. On the other hand, in a qualitative study from Norway about GPs and CPs positioning each other in relation to power dynamics, the GPs stated the CPs as “unknown”, and the CP stated the GPs as “on top of the hierarchy” [6, 7]showing that interprofessional collaboration is not self-evident in day-to-day practices. In a systematic review, Weißenborn et al. identified six indicators for a successful collaboration, such as personal contact, and the authors suggest that interprofessional education could be the first step towards a trusting longtime relationship [8]. Because of the positive effects of interprofessional collaboration the ‘exposure’ to the topic should start as early as possible in the career [4]. Thus, the implementation of interprofessional education is necessary and demanded by different organizations [4, 9, 10]. The Centre for the Advancement of Interprofessional Education defines interprofessional education as “when two or more professions learn from and about each other to improve collaboration and the quality of care”. [11]^(p6)^ There are a lot of interprofessional education projects established around the world with healthcare professionals from different disciplines [12–19]. However, a controlled study design is rarely used and there are not many interprofessional education programs in Germany especially with medical and pharmacy students [20–22]. 

The aim of our pilot study was to assess whether the “interprofessional collaboration between medical and pharmacy students to improve medication safety in polypharmacy” (PILLE) project changed the perception of medical and pharmacy students towards interprofessional collaboration.

## Methods

### Study design and participants

PILLE consisted of three parts: (1) A mandatory 90-minutes interprofessional seminar; (2) A voluntary 120-minutes practical training; (3) A voluntary half-day interprofessional tandem work shadowing at a GP’s office. The development of the concept and the initial phase have been described in detail [23]. The pilot study was carried out in cooperation between the Institute of General Practice and Family Medicine of the Charité– Universitätsmedizin Berlin and the Institute of Pharmacy of Freie Universität Berlin from May 2022 to June 2023. We investigated the change of perception after participating in the PILLE project.

Two seminar groups of the medical students from the 10th semester (out of 12) of the integrated, competency-based Modular Curriculum of Medicine (MCM) [24] were part of the PILLE intervention. The medical students from other seminar groups were used as control group. All pharmacy students from the 7th semester (out of 8) within the discipline of “Clinical Pharmacy” of the Pharmacy Staatsexamen-Curriculum participated in the interprofessional education project, therefore it was not possible to form a control group. Medical and pharmacy students from the intervention group attended the mandatory interactive 90-minutes interprofessional seminar (1). After inputs and discussions about medication safety and multimorbidity in the first half of the seminar, the medical and pharmacy students prepared the case in small interprofessional groups for the plenum discussion in the second half of the seminar. Medical students of the control group attended an interactive 90-minutes monoprofessional seminar only. The medical students prepared the case in small monoprofessional groups for the plenum discussion at the end of the seminar. Thus, working together on cases was the similarity of the mono- and interprofessional seminars, using the same case. In the monoprofessional seminar some lecturers decided to use a second case. The students from the intervention group could voluntarily attend the interprofessional 120-minutes practical training with more case work (2). Additionally, the pharmacy students could accompany the medical students within their two-weeks work shadowing in a GP’s office for a half day (3).

### Data collection

The outcome of interest was the change in students’ perception towards interprofessional collaboration after participating in the interprofessional education project PILLE, using the validated German version of Student Perceptions of Physician-Pharmacist Interprofessional Clinical Education (SPICE-2D) instrument [25]. Pre-questionnaires were sent via e-mail before the seminar to intervention and control group. Post-questionnaires were sent via e-mail to all students of the intervention group after the timeframe of the two-weeks work shadowing in a GP’s office ended and to the control group directly after the seminar. The SPICE instrument was developed by Fike et al. [26] and refined by replacing three items by Zorek et al. (SPICE-2) [27]. Pudritz et al. translated the SPICE-2 instrument to German (SPICE-2D) [25]. The SPICE-2D instrument measures perceptions of interprofessional collaboration as overall score and consists of 10 items from 3 factors (teamwork and team-based practice, roles/responsibilities for collaborative practice, and patient outcomes from collaborative practice) using a 5-point Likert scale. Higher values demonstrate a more positive perception [25–27]. The students gave themselves an ID, so that the pre- and post-questionnaire were relatable for anonymised data analysis. We also conducted qualitative interviews which are analysed separately.

### Data analysis

Descriptive and statistical evaluation was conducted using Microsoft^®^ Excel^®^ (Microsoft 365, Redmond, Washington, USA) and SPSS^®^ Version 29.0 (IBM Corp, Armonk, New York, USA). Data are presented as mean ± standard deviation (SD), pre-post-mean-difference and 95% confidence intervals. Normal distribution was assessed graphically using Q-Q-Plots. To assess if there is a significant pre-post-difference in the SPICE-2D overall score within the individual groups, paired t-tests were executed. To investigate if there is a significant change in SPICE-2D overall score difference between the medical students control and intervention group, an unpaired t-test was executed. Non-parametric testing within the groups (Wilcoxon signed-rank test) and between the medical students control and intervention group (Mann-Whitney U-test) were conducted to back up the results from parametric testing, leading to the same conclusion. The significance level in all tests was *alpha* = 0.05.

### Ethics

The students gave their written informed consent, and the pilot study was approved by the Ethics committee at the Charité– Universitätsmedizin Berlin (EA1/103/22).

## Results

### Sample

In total, 436 students participated: 96 medical students and 116 pharmacy students in the intervention group, and 224 medical students in the control group. In the voluntary part, 30 pharmacy and 10 medical students (intervention group) worked together on cases and only 10 students reported participating in the self-organized interprofessional tandem work shadowing at a GP’s office. Table 1 shows the overall demographics of the cohorts.

**Table 1:** Demographic data of medical students of the control group and of medical and pharmacy students from the intervention group

Demographic data	Medical students control group N (%)	Medical students intervention group N (%)	Pharmacy students intervention group N (%)
Participants: seminar	224 (100%)	96 (100%)	116 (100%)
Participants: practical training	NA	10 (10%)	30 (26%)
Participants: work shadowing in a GP’s office*	NA	6 (6%)	4 (3%)
Pre-Questionnaire**	112 (50%)	72 (75%)	81 (70%)
Post-Questionnaire**	55 (25%)	25 (26%)	49 (42%)
Pre-Post-Responses**	47 (21%)	23 (24%)	39 (34%)
Sex			
Female	30 (64%)	14 (61%)	25 (64%)
Male	17 (36%)	9 (39%)	14 (36%)

*self-organized, reported in post-questionnaire **including SPICE-2D, NA: not applicable, GP: general practitioner

### Change in perception

The participating students from both professions already had a pronounced positive perception at baseline: SPICE-2D overall scores were 38.4–39.7 (maximum possible score: 50). The pre-post-mean-difference was numerically larger at the end of the project, but the differences were not statistically significant (Table 2).

**Table 2:** SPICE-D overall score

	Pre-Questionnaire	Post-Questionnaire	Pre-Post-Mean-Difference	*p*-value*
	Mean [SD]	Mean [SD]	[95% CI]	
**Overall (score: 10–50)**				
Medical students control	38.4 [4.1]	38.7 [4.4]	0.3 [-0.7; 1.2]	0.561
Medical students intervention	38.4 [3.0]	39.5 [3.9]	1.1 [-0.5; 2.6]	0.177
Pharmacy students intervention	39.7 [4.8]	40.5 [5.1]	0.8 [-0.4; 2.0]	0.209

*paired t-test, p-value < 0.05, SD: standard deviation, 95% CI: 95% confidence interval

The pre-post-mean-difference in the medical students control group was numerically lower compared to the medical students of the intervention group, but there was no statistical significance (unpaired t-test, *p* = 0.372).

## Discussion

### Summary of the results

In the PILLE project, there was no significant overall pre-post-difference in perception towards interprofessional collaboration; neither compared to the control group nor within the intervention groups. The students already had a pronounced positive perception before participating, therefore PILLE had no effect on perception.

### Comparison to other interprofessional education projects

The positive perception towards interprofessional collaboration in this pilot study is in line with findings of other projects, e.g. the “POP-Art” project. In this project, medical and pharmacy students joined an interprofessional bedside teaching in the hospital with comparable SPICE-2D-results. In addition, the pharmacy students had also higher pre- and post-SPICE-2D scores than the medical students [21]. Our results also fit to the evaluation of an interprofessional high-fidelity simulation course on medication therapy consultation where all participants had a positive attitude towards interprofessional collaboration and training. This study additionally assessed the students’ performance using objective structured clinical examination (OSCE), where a significant difference in performance was shown. The interprofessional training group performed better [20]. Nevertheless, other projects using SPICE-R2 (version for all health professions students) or SPICE-2 (English version) showed a significant difference in pre-post overall score. In contrast to our pilot study, these studies were longer, more interactive, workplace-based and/or voluntary [19, 28–30]. 

### Structural barriers to interprofessional education

Merging students’ curricula with fixed schedules from two different organizations with learning facilities far away from each other, was challenging in our study. But despite the different curricula, timetables and locations it was possible to make the seminar mandatory, which contrasts with other projects often being voluntary. The PILLE project also left project status, which is also different from other projects [31]. A challenge was the limited time within the small groups and plenum to focus on interprofessional competencies, e.g. communication or teamwork. The focus was on medication safety, multimorbidity and solving the drug-related and medical problems of the case work instead. Within the other two parts the focus was also on interprofessional competencies [32–34]. Because of organizational barriers the other two parts were voluntary, so only a few students participated. In addition, the students might have too different professional mindset and heterogenous learning cultures, making it challenging to learn from each other in the short time available. This has been shown and discussed in other projects [34–37]. The 90-minutes seminar as the only mandatory part, was too short to measure a change in perception. In an across semester evaluation from the Charité– Universitätsmedizin Berlin, the medical students rated the relevance of interprofessional collaboration as (very) important, but only around one third perceived a (very) large coverage in the curriculum [38]assuming that the students’ positive perception in our project is not from other interprofessional education experiences. Curriculum changes might be necessary to have sustainable effects, e.g. having joint modules or longer joint experiences to minimize challenges, also described in other publications [31, 34, 35]. In our study an above-average committed interprofessional team, including a medical and pharmacy student, developed the PILLE project. The importance of a committed leadership team and the participation of students in the working group have also been shown in other studies [31, 34, 35, 39]. 

### Strengths and weaknesses of the study

Strengths of this pilot study are the study design by using a control group for the medical students and the concept itself. Another strength is the use of a validated evaluation instrument.

A weakness is that there were no pharmacy students available to form a control group because all pharmacy students in Berlin attended the interprofessional education project. The drop in participants response after the project is typical for all teaching evaluations in Berlin, as these are not allowed to be mandatory. In addition, only the participants who filled out the pre- and post-questionnaire could be used for evaluation, minimizing the sample size. Therefore, the sample size was too small to show an effect; however, a power and intervention effect and, hence, sample size calculation was not feasible. A weakness of the questionnaires was the missing question about possible students’ previous interprofessional education experience with other health care professionals. Students with already a positive perception towards interprofessional collaboration were potentially more likely to fill-in both questionnaires, especially in the control group (selection bias). In addition, the students might not be aware of all dimensions of collaboration, when answering the pre-questionnaire, leading to a ceiling effect [40]. The use of a post-pre-post-questionnaire like Interprofessional Collaborative Competency Attainment Survey (ICCAS) might have increased the response rate and minimized the ceiling effect, because of only filling out one questionnaire after participating in the project [41]. Instead of perceptions, another potential outcome measure could have been the actual performance of students. Sharkas et al. used the method of OSCE in an intervention study [20]. 

## Conclusions

Participating medical and pharmacy students already had a positive perception towards interprofessional collaboration at baseline. PILLE resulted in a numerical increase of the SPICE-2D overall score, but no observed differences were significant. A one-time and short-term mandatory seminar of 90-minutes seems not to be sufficient to measure a change in perception in students with already positive perceptions. Future interprofessional education projects should focus on students with lower perceptions towards interprofessional collaboration and on frequent meeting points between students. Furthermore, the measurement of performance could be a suitable endpoint for interprofessional education studies.

## Data Availability

The datasets generated and/or analysed during the current study are not publicly available but are available from the corresponding author on reasonable request.

## Abbreviations

CI: Confidence interval
CP: Community Pharmacist
GP: General Practitioner
ICCAS: Interprofessional Collaborative Competency Attainment Survey
OSCE: Objective structured clinical examination
SD: Standard deviation
SPICE: Student Perceptions of Physician-Pharmacist Interprofessional Clinical Education
